# Intra-Articular Corticosteroid Injection After Total Knee Replacement: Is it Safe?

**DOI:** 10.7759/cureus.19700

**Published:** 2021-11-18

**Authors:** NagaSuresh Cheppalli, Naveen Singanamala, Timothy J Choi, Ashish Anand

**Affiliations:** 1 Orthopedics, Veteran Affairs Medical Center (VAMC), Albuquerque, USA; 2 Orthopedics and Rehabilitation, University of New Mexico School of Medicine, Albuquerque, USA; 3 Orthopedics, St. Barnabas Hospital Health System, Bronx, USA; 4 Orthopedics, University of New Mexico School of Medicine, Albuquerque, USA; 5 Orthopedics, Veteran Affairs Medical Center (VAMC), Jackson, USA; 6 Orthopedics, University of Mississippi Medical Center, Jackson, USA

**Keywords:** knee corticosteroid injection, periprosthetic joint infection, total knee arthroplasty, total knee replacement, intra-articular steroids

## Abstract

Recalcitrant pain after total knee replacement (TKR) is sometimes treated with intra-articular steroid injections (IASI), with few studies reporting on the risk of subsequent periprosthetic joint infection (PJI). This is a systematic review to evaluate the incidence and risk of PJI after IASI into a total knee replacement.

We searched online databases using the keywords "total knee replacement," "total knee arthroplasty," "steroids" and "intra-articular injection." A total of 7386 articles (PubMed - 91, Embase - 70, Web of Science - 57, CINAHL - 8, and Google Scholar - 7160) were retrieved on the initial search. After applying exclusion criteria, four articles were included in this review for evaluation and statistical analysis. There were no level one or two studies.

The incidence of infection after IASI at 12 months was 138/6499 or 2.1%, while the incidence of infection rate among controls at 12 months was 158/11256 or 1.4%. A chi-square test showed that the difference in infection rate was significant (p = 0.0002424). A caveat is that simple statistical test results are virtually guaranteed to be statistically significant with large sample size.

IASI into a TKR is not a benign procedure and that may be associated with a significantly increased risk of subsequent periprosthetic joint infection. We, therefore, recommend against IASI into a TKR until better studies can be performed to determine their safety and efficacy.

## Introduction and background

Introduction

Total knee replacement (TKR) is a commonly done procedure in end-stage osteoarthritis of the knee. It can improve the patient's symptoms and lifestyle significantly [1]. Despite advances in technology, there is a proportion of patients who remain dissatisfied after surgery, secondary to pain or stiffness, varying from 13-54% [2-6]. The etiology of chronic pain after TKR is multifactorial. It can limit patients' functional capacity and demands a thorough workup. The possible causes include infection, aseptic loosening, or periprosthetic fracture, instability, component malposition, soft tissue irritation, metal allergy, or a combination of any of these above [7-9]. Management of most of these problems includes revision surgery. The patients with defined causes fare better than those whose workup is negative [10]. Therefore, there remains a subset of patients who, despite extensive workup, do not have an apparent cause of pain after TKR, and they remain unhappy after the procedure [11-13].

Based on classical teaching, physicians have been reluctant to use intra-articular steroid injections (IASI) in either total or partial knee replacement due to the increased risk of periprosthetic joint infection (PJI), a potentially catastrophic event. However, there is conflicting evidence to support this point. A few studies have reported their use in recent times [14-16]. This is a systematic review to evaluate the incidence and risk of periprosthetic joint infection (PJI) after intra-articular steroid injections (IASI) after total knee replacement (TKR). We also evaluated the quality of included studies using methodological index for non-randomized studies (MINORS) scale in this review [17].

Materials and methods 

Search Strategy

Two independent reviewers performed systematic research using Preferred Reporting Items for Systematic Reviews and Meta-Analyses (PRISMA) guidelines. We searched Medline, Embase, Google Scholar, Cumulative Index to Nursing and Allied Health Literature (CINAHL), Web of Science, Cochrane database using the keywords "total knee replacement," "total knee arthroplasty," "steroids" and "intra-articular injection." The same keywords were used in Google Scholar to search for other studies not published in PubMed indexed journals. We included all the published studies in English literature showing IASI after TKR to manage pain and/or stiffness in the knee. 

We excluded all studies reporting pre-operative IASI and peri-operative steroid injections as part of multimodal analgesia. We also excluded studies of non-corticosteroid injections into TKR and pericapsular corticosteroid injection after TKR for treating residual pain (Table 1).

**Table 1:** Inclusion and exclusion criteria TKR: total knee replacement; IASI: intra-articular steroid injections

Inclusion Criteria	Exclusion Criteria
Intra-articular corticosteroid injection after TKR	Pre-operative IASI in knee joint, peri-operative steroid injection (as a part of multimodal analgesia), peri-articular/peri-capsular injections at knee joint after TKR for treating residual pain

Data Abstraction

**Figure 1:**
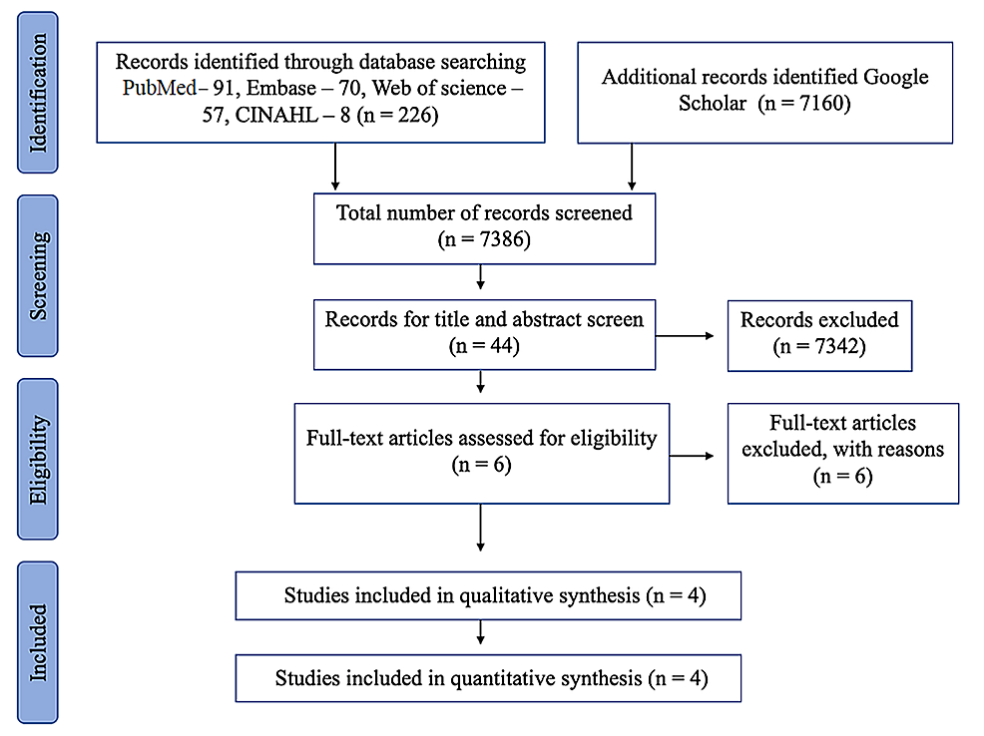
PRISMA follow chart showing literature search and methodology of selection CINAHL: Cumulative Index to Nursing and Allied Health Literature; PRISMA: ​​​Preferred Reporting Items for Systematic Reviews and Meta-Analyses

A total of 7386 (PubMed - 91, Embase - 70, Web of Science - 57, CINAHL - 8, Google Scholar - 7160) articles were retrieved on the initial search. We included only the articles that have used intra-articular steroid injections (IASI) to manage residual pain after TKR. Forty-four articles were initially selected based on information from the titles and their abstracts were reviewed. We only had six articles related to this topic after excluding duplicates, articles on pre-operative and peri-operative corticosteroid injections. We downloaded full-text articles of these six articles. After review, we excluded two articles, as they had described peri-operative administration of corticosteroids as a part of multimodal analgesia followed by radiofrequency ablation [18] and pes anserinus bursal injection [19]. We also screened all the references from fully retrieved articles not to miss any relevant studies. We noted one more article, but it had not described the duration of follow-up, so we excluded this study as well [20]. Finally, a total of four articles were included in this review for evaluation and statistical analysis, summarized in Table 2. Two independent (NC and AA) reviewers did all these steps (Figure 1). We assessed the methodological quality of the studies retrieved for this systematic review. Two reviewers (NC and NS) independently performed appraisal bias using the MINORS scale, scored out of 24 (Table 3) [17]. There were no level one or two studies available for review. 

**Table 2:** Summary of included articles PJI: prosthetic joint infection; ICD: International Classification of Disease; ROM: range of motion; MUA: manipulation under anesthesia

Author	Number of patients	Number of surgeons	Infection rate	Definition of infection	Follow-up (months)	Effectiveness	Workup	Type of steroid	Place of administration	Level of evidence	Time from injection to PJI (months)	Conclusion
Millis et al. [15]	736	Multiple surgeons	0.4%	PJI	37	Not determined	None	DepoMedrol/Kenalog	Clinic	IV	22.66	Could not establish relation between infection and steroid
Klement et al. [21]	184	Single surgeon	0%	Clinical evaluation	12	Yes	Yes	Kenalog 40mg	Clinic	IV	No Infection	Steroid injection can result in symptomatic improvement
Roecker et al. [16]	5628	Multiple surgeons	1.9%	ICD classification	12	Not determined	N/A	N/A	N/A	III	N/A	Significant association with infection
Sharma et al. [14]	6	N/A	0%	Clinical	14.6	Attained better ROM	N/A	N/A	Operating Room	IV	No Infection	Improves ROM after MUA

**Table 3:** Quality appraisal using MINORS table The items are scored 0 (not reported), 1 (reported but inadequate), or 2 (reported and adequate). The global ideal score is 16 for non-comparative studies and 24 for comparative studies. MINORS: methodological index for non-randomized studies

Criteria	Millis et al. [15]	Klements et al. [21]	Roecker et al. [16]	Sharma et al. [14]
1. A clearly stated aim: the question addressed should be precise and relevant in the light of available literature	2	2	2	2
2. Inclusion of consecutive patients: all patients potentially ﬁt for inclusion (satisfying the criteria for inclusion) have been included in the study during the study period (no exclusion or details about the reasons for exclusion)	2	2	2	2
3. Prospective collection of data: data were collected according to a protocol established before the beginning of the study	3	2	0	2
4. Endpoints appropriate to the aim of the study: unambiguous explanation of the criteria used to evaluate the main outcome which should be in accordance with the question addressed by the study. Also, the endpoints should be assessed on an intention-to-treat basis.	2	2	2	2
5. Unbiased assessment of the study endpoint: blind evaluation of objective endpoints and double-blind evaluation of subjective endpoints. Otherwise, the reasons for not blinding should be stated	0	0	0	0
6. Follow-up period appropriate to the aim of the study: the follow-up should be sufﬁciently long to allow the assessment of the main endpoint and possible adverse events	2	2	2	2
7. Loss to follow-up less than 5%: all patients should be included in the follow-up. Otherwise, the proportion lost to follow-up should not exceed the proportion experiencing the major endpoint	2	2	2	2
8. Prospective calculation of the study size: information of the size of detectable difference of interest with a calculation of 95% conﬁdence interval, according to the expected incidence of the outcome event, and information about the level for statistical signiﬁcance and estimates of power when comparing the outcomes	0	0	2	0
Additional criteria in the case of comparative study				
9. An adequate control group: having a gold standard diagnostic test or therapeutic intervention recognized as the optimal intervention according to the available published data			1	2
10. Contemporary groups: control and studied groups should be managed during the same time period (no historical comparison)			2	2
11. Baseline equivalence of groups: the groups should be similar regarding the criteria other than the studied endpoints. Absence of confounding factors that could bias the interpretation of the results			2	2
12. Adequate statistical analyses: whether the statistics were in accordance with the type of study with the calculation of conﬁdence intervals or relative risk			2	2
Total score	13	12	19	20

Results

**Figure 2:**
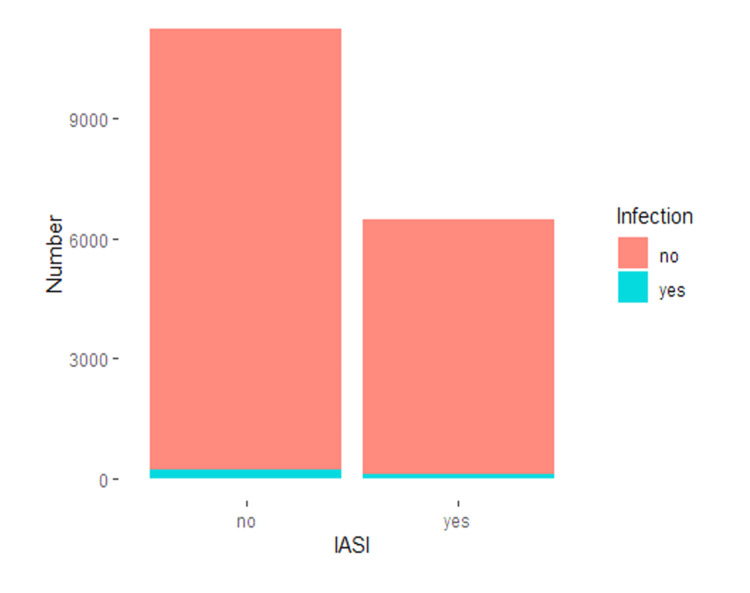
The left column represents the total number of subjects after TKR without IASI (controls) and the right column represents subjects with IASI The blue bar represents infection and the orange bar represents no infection group. TKR: total knee replacement; IASI: intra-articular steroid injections

Periprosthetic joint infection (PJI) rate after IASI was the primary outcome evaluated in this review. The number of patients and reported infections from all the articles were added together for statistical analysis. The IASI group's incidence of infection at 12 months was 138/6499 or 2.1%, while the incidence of infection rate among the controls at 12 months was 158/11256 or 1.4%. As we did not have access to the studies' raw data, we performed a chi-square test on the numbers. We found that the difference in infection rate was significant (p = 0.0002424). A caveat is that simple statistical test results are virtually guaranteed to be statistically significant with a sample size as large as the one considered (Table 4, Figure 2).

**Table 4:** Statistical analysis of included articles IASI: intra-articular steroid injections; TKR: total knee replacement; OR: odds ratio; CI: confidence interval

Author	Number of patients who received IASI	Infections	Follow-up (months)	Pre injection work	Time after TKR (months)	OR with CI	Control
Mills et al. [15]	736	3	37	No	51	N/A	None
Klement et al. [21]	129	0	12	Yes	5.3	N/A	None
Sharma et al. [14]	6	0	14.6	N/A	2	N/A	Yes
Roecker et al. [16]	5628	107	12	N/A	N/A	OR, 1.85; 95% CI, 1.54-2.21; p <0.0001	Yes

## Review

Critical appraisal

In a series of 2015 patients receiving IASI into aTKR, Mills et al. presented 1845 injections in 736 patients with a mean follow-up of 37 months (range: 3-90 months) [15]. They reported PJI in three patients within three months of the intra-articular steroid injection, yielding a PJI rate of 0.16% or one PJI per 625 injections (three infections in 736 patients, i.e., 0.4% of patients injected had infections). The authors did not report routine workup to rule out infection in TKR before IASI administration.

One of the infected patients had no workup to rule out an infected TKR before corticosteroid injection into that knee. The second patient had five intra-articular corticosteroid injections into a TKR over two years and developed a PJI after the sixth injection. The third patient had a corticosteroid injection into a painful TKR three years after the index surgery and two months later had a genicular nerve ablation. This patient developed a PJI 24 hours after the nerve ablation. The authors also report that four other patients developed PJI after IASI into a TKR, which occurred six to 53 months after IASI and were considered unrelated to the injections. Overall, the authors do not infer the causation of PJI in their patients and only report the correlation with IASI after TKR. The authors did not rule out infection before IASI, a potential cofounder in this study, as was the absence of a matched control group of patients who had the TKR but not the injection. 

A retrospective study by Klement et al. examining the efficacy of IASI after TKR in 184 patients concluded that there were zero instances of PJI within 12 months, while most patients had some improvement of pain after the injection [21]. They also reported high patient satisfaction with the injections. The patients were evaluated clinically and had a laboratory (ESR, CRP) and knee aspiration if the preceding were abnormal, and radiographic evaluation to rule out infection before the corticosteroid injection into the TKR. Patients were also assessed by a mailed survey four weeks after the injection and then called (after a second mailed survey went unanswered), with a final response rate of 129/184 patients (70.1%). Overall, 184 patients received at least one IASI into a TKR, at an average of 5.3 months after TKR. Of these, 30.8% of patients received more than one injection (range: 1-5). The patients reported that pain decreased (in 76.6%), increased range of motion (57.9%), and reduced swelling (65.4%). Adverse events included pruritus, pain, swelling, clicking, and/or decreased energy in six patients (5.6%). Strength of this study was the pre-injection evaluation to rule out infection. The authors ruled out PJI based on a complete chart review of all injected patients and cross-referenced it with an institutional joint replacement database. Lack of a reported clinical evaluation of the TKRs after the injections during the study period of 12 months is a prominent weakness of this study, as is the lack of a matched control group. The authors concluded that IASI after TKR was a viable option for treating a painful TKR in the absence of infection, with an excellent subjective improvement of pain and function and minimal side effects. Other drawbacks of the study were that only 82.9% of injected patients responded to the questionnaire and the potential for recall bias and the absence of a matched control group of TKR patients who had not received the injections.

In the largest study to date, a national database (Humana dataset from the Pearl Diver Database) was analyzed by Roecker et al., who looked at 166,946 TKRs, of which, 5628 patients had an intra-articular corticosteroid injection after surgery (3.4%) [16]. They were matched 2:1 for multiple co-morbidities, age, and sex, with patients who underwent TKRs but not post-operative steroid injections into ipsilateral knees. The data were analyzed to determine periprosthetic joint infection rates at six months and one year after the injections and knee infections up to one year pre-operatively. They assessed the incidence according to the International Classification of Disease (ICD) 9 and ICD 10 codes for PJI and matched them with CPT codes for surgery for PJI and with laterality codes to ensure that the same knees that had the IASI also had the PJI and the surgery for it. The results indicated that within six months after IASI into a TKR, 1.9% of TKRs became infected (107 patients) versus 1.1% in the control group. This difference was found to be statistically significant (OR, 1.85; 95% CI, 1.54-2.21; p <0.0001) after matching for demographics and co-morbidities. Similarly, one year after post-operative corticosteroid injection, these patients had an increased infection rate of 2.4% versus 1.4% in controls. This difference was also statistically significant (OR, 1.97; 95% CI, 1.64-2.36; p <0.0001). Overall, at any post-injection time point, study patients had a significantly higher periprosthetic infection rate in the injected TKR at 1.8%, compared to 0.4% in controls (no injection). This difference was also statistically significant (OR, 7.36; 95% CI, 5.47-9.91; p <0.0001). This study has the advantage of having a large number of patients in the matched control and injection groups, which improved the power and validity of statistical correlation. It is also the most systematic study that has been published to date. Drawbacks of this large database study include its retrospective nature and possible coding errors that may limit the data's accuracy. Besides, pre-injection workup to rule out infection could not be established. Surgeon-specific criteria for infection and indications for injection remained unknown. The authors did not report if the injections helped or not. The other potential confounding factor is that as the IASI is given in painful TKR and the possibility of undiagnosed infections could be higher in this group.

Sharma et al. in a retrospective review reported that six TKRs (out of 292 TKRs in the "injection" group) were injected with 0.5% bupivacaine (200-400mg), morphine (8mg), epinephrine 1 in 1000 (300mcg), methylprednisolone (40mg), cefuroxime (750mg), and 0.9% NaCl (42cc) after manipulation under anesthesia for post-operative stiffness [14]. This group also had intra-operative peri-articular steroid injections as part of a multimodal analgesic regimen at the time of the index TKR. The "control" group had 286 TKRs that had no intra-operative peri-articular steroid injection and had seven TKRs manipulated without any intra-articular injection at the time of manipulation. The mean time for manipulation after TKR in the injection group was 9.6 weeks (range: 7-13 weeks). They reported no infections and no re-operations at a maximum follow-up of 18 months (mean = 14.6 months), which was similar to the "control" group, which received no peri-articular intra-articular injections at index surgery or after manipulation. It is possible that the addition of a broad-spectrum antibiotic, cefuroxime, to the intra- and post-operative injections played a role in the mitigation of infection risk after injection in the study group, which is a distinguishing feature of this study [20,21].

Discussion

IASI after TKR is not a benign procedure and may be associated with an increased rate of PJI. The risk of infection following IASI is extremely low in the native knee and can be around 1 in 14,000 to 15,000 (0.007%) [22]. There have been rare instances of septic arthritis associated with IASI done in the native knee [23]. Some studies reported an increased risk of PJI in total knee arthroplasty (TKA) and total hip arthroplasty (THA) with pre-surgical (three months before TKR) IASI [24,25]. But, peri-operative steroid injection during TKR as part of multimodal pain management has been shown to provide additional pain control without an increased risk of PJI [26]. Some meta-analyses have revealed no increased risk of PJI following IASI administration before TKR [27]. The role of intra-articular steroid administration in a knee joint in the development of infection is debatable. Even though IASI for managing knee pain is a common practice, the practice of administering steroid injection for managing residual pain after TKR injections is uncommon (about 3.4% in a large database study), for the feared risk of infection [16]. The increased rate of infection might be due to immunosuppression, or contamination of joints by the injection process due to varied practices regarding antiseptic technique. Despite this existing controversy, various authors have reported on the use of IASI as a pain-relieving intervention for painful or stiff TKR [14-16,19]. Some surgeons used IASI either alone or in conjunction with antibiotics after manipulation under anesthesia with no resultant increase in infection, with an increase in range of motion (ROM) in the group with IASI [14].

Considering the patient's autonomy in treatment selection, the invasive nature of the procedure, and the low rate of post-operative IASI and knee infection after IASI, it would be difficult to conduct an adequately powered randomized control trial. In such a case, a systematic review such as the current study with analysis of data pooled from the available studies could provide good evidence. As stated earlier, the incidence of infection in the IASI group was 2.1% (138/6499), while the incidence of infection in the control group was 1.4% (158/11,256). We found that the difference in infection rate was significant (p=0.0002424). The controls were derived from one study by Rocker et al. (2:1 matched for co-morbidities from Humana database) [16]. The increase in the absolute risk of infection attributed to IASI was 0.7% when compared to their control group.

This study is not without any limitations. This current review is a systematic analysis of four different studies. As Rocker et al. had the most number of subjects (86% of total subjects), this study had the maximal effect on the results [16]. Each study included in this review had a different population and protocols, some including a pre-injection workup for infection. Due to this heterogeneity in subjects, study methodologies, and lack of prospective randomization, the results of our review should be interpreted with caution.

Based on the current evaluation, it is imperative to rule out PJI while evaluating painful TKR before offering any intervention, including injection. The indications for IASI were also not mentioned. There is no data on the aseptic precautions, location of administration (office setting or operating room, minor procedure room), type of needle (hollow vs. solid bore needle), and the kind of steroid (particulate vs. non-particulate) used from these studies, all of which could influence the outcome. Klements et al. attempted to evaluate the efficacy of IASI in relieving pain [21]. However, even this study has many methodological flaws, including recall bias and 30% loss to follow-up. In his study, he concluded that IASI could be a viable option after TKR to manage residual pain and did not report any infections in this group. Patients from this study reported decreased pain (in 76.6%), increased range of motion (57.9%), and reduced swelling (65.4%). Adverse events included pruritus, pain, swelling, clicking, and/or decreased energy in six patients (5.6%). No infection is reported from this study. The strength of this study is pre-operative work up to rule out infection, absent from the other studies. 

When non-database studies were evaluated, a total number of 926 patients received IASI and reported only three infections (3/926=0.32%) [15,21,14]. This rate of infection is much lower than the controls from the database study. This observation raises the other following questions: is the medicare population (from the Humana database) more prone to infection than others? Is there a possibility of "over-reporting" of PJI in database studies (due to coding differences, criteria for the diagnosis of infection, etc.) and under-reporting in others? Interpretation of this database study with the non-database studies leads to a question of whether steroid injections into pre-existing TKRs are riskier in some groups of the population than others. The Humana database study we reviewed had most patients above 65 years of age [16]. Unfortunately, the authors of the other studies did not report the age.

Variations in aseptic preparation before administering the steroid injections could change the risk of the introduction of infection. In current practice, there is no uniformity regarding aseptic preparations during these procedures and is still not conclusively known whether the effect of the steroid in the joint or the injection procedure itself (which can introduce skin contaminants into the joint) is the culprit.

The effectiveness of these injections has also not been fully elucidated. Only one study attempted to evaluate the IASI's effectiveness for managing post residual pain of the TKR, and even that had significant recall bias and no control group [21]. Although not included in our systematic review, PJI has been reported post-TKR after receiving acupuncture, genicular nerve ablation, and peri-articular injections [15,18]. Therefore, as the effectiveness of intra-articular steroid injection was not adequately demonstrated in a painful TKR and because of the association of a significantly increased risk of PJI after IASI, we recommend against IASI after TKA until better studies can be performed to determine their safety and efficacy.

We hope that future research will answer the following seven questions: (1) Are intra-articular steroid injections effective in treating post-TKR residual pain? If yes, how long after index surgery are they best administered? What are the indications? How long does the benefit last? (2) If IASI after TKR has an increased risk of infection, what are the circumstances and patient characteristics? (3) Are infections due to the introduction of skin contaminants through injections or the ostensibly powerful (due to high local concentration) local immunosuppressive action from steroids? (4) If effective, what is the best corticosteroid used for managing post residual pain? Is there a difference in particulate versus non-particulate preparations? (5) Does the inclusion of an antibiotic in the IASI change the infection risk? (6) What are the best place, time (duration from surgery), and practices to perform such injection procedures? (7) Does a mandatory pre-injection work-p to rule out infection change the outcome?

## Conclusions

There is a lack of reliable studies on the indications, utility, and incidence of PJI after IASI after TKR. There may be some value in treating post-TKR residual pain using IASI, especially once infection and surgically correctable causes for painful TKR are ruled out. However, based on current information, we conclude that IASI in TKR is not a benign procedure and that it may be associated with a significantly increased risk of subsequent periprosthetic joint infection. We, therefore, recommend against IASI after TKR until better studies can be performed to determine their safety and efficacy.
